# Supplementary material for: Enzyme adaptation to habitat thermal legacy shapes the thermal plasticity of marine microbiomes
Source: Nat Commun. 2023 Feb 24;14:1045. doi: 10.1038/s41467-023-36610-0 (PMC9958047; doi:10.1038/s41467-023-36610-0)
Supplement: Supplementary file 1 — Supplementary Information [file 41467_2023_36610_MOESM1_ESM.pdf]

SUPPLEMENTARY INFORMATION for

Enzyme adaptation to habitat thermal legacy shapes the plasticity of marine microbiomes

Ramona Marasco[1#], Marco Fusi[1,2#], Cristina Coscolín[3#], Alan Barozzi[1], David Almendral[3], Rafael Bargiela[4], Christina Gohlke neé Nutschel[5], Christopher Pfleger[6], Jonas Dittrich[6], Holger Gohlke[5-7], Ruth Matesanz[8], Sergio Sanchez-Carrillo[3,9], Francesca Mapelli[10], Tatyana N. Chernikova[4], Peter N. Golyshin[4], Manuel Ferrer[3]*, Daniele Daffonchio[1]*

[1]Biological and Environmental Sciences and Engineering Division (BESE), Red Sea Research Centre (RSRC), King Abdullah University of Science and Technology (KAUST), Thuwal 23955-6900, Saudi Arabia
[2]Edinburgh Napier University School of Applied Sciences, Sighthill Campus, Sighthill Court Edinburgh EH11 4BN, United Kingdom
[3]ICP, CSIC, Marie Curie 2, 28049 Madrid, Spain
[4]Centre for Environmental Biotechnology, School of Natural Sciences, Bangor University, Deiniol Rd, Bangor, LL57 2UW, United Kingdom
[5]Institute of Bio- and Geosciences (IBG-4: Bioinformatics), Forschungszentrum Jülich GmbH, 52425 Jülich, Germany.
[6]Mathematisch-Naturwissenschaftliche Fakultät, Institut für Pharmazeutische und Medizinische Chemie, Heinrich-Heine-Universität Düsseldorf, 40225 Düsseldorf, Germany.
[7]John von Neumann Institute for Computing (NIC) and Jülich Supercomputing Centre (JSC), Forschungszentrum Jülich GmbH, 52425 Jülich, Germany.
[8]Spectroscopy Laboratory, Centro de Investigaciones Biológicas Margarita Salas, CSIC, Madrid 28040, Spain
[9]Severo Ochoa Molecular Biology Center, Consejo Superior de Investigaciones Científicas (CSIC), Madrid 28049, Spain.
[10]Department of Food Environmental and Nutritional Sciences, University of Milan, Milan 20133, Italy

[#]Equal contribution

*Correspondence to: Daniele Daffonchio (daniele.daffonchio@kaust.edu.sa) and Manuel Ferrer (mferrer@icp.csic.es)

Running title: Thermal legacy tunes microbial community plasticity

SUPPLEMENTARY NOTES

Supplementary Note S1. Sequence similarity of the 78 esterases from the Iris Sea, Mediterranean, and the Red Sea is reported in Supplementary Figure S8. According to sequence similarities and the presence of conserved motifs[1], these esterases included sequences with a typical α/β hydrolase fold and conserved G-X-S-X-G motif (Family [F]I=6; FIV=35; FV=18; FVI=3; FVII=2; FX=2), one sequence belongs to the true lipase sub-family, one sequence shows a serine beta-lactamase-like modular (non-α/β hydrolase fold) architecture and a conserved S-X-X-K motif (FVIII), and an additional set of nine sequences were assigned to the meta-cleavage product hydrolase (MCPH) family with typical α/β hydrolase fold. All the 150 esterases from Tara Ocean belong to FIV. Pairwise sequence similarity among all sequences reveals low values for both datasets: 16.7% for the sequences retrieved from the Irish Sea, Mediterranean and the Red Sea, and 38.1% for those obtained from the Tara Ocean.

Supplementary Note S2. Bacterial OTUs distribution as a function of sediment temperatures. While 17% of OTUs (n = 1041 OTUs; 10% of relative abundance) were negatively correlated with increasing temperature, 1% of OTUs (n = 50 OTUs; 6% of relative abundance) increment with them (Supplementary Data S5). Among these, the OTUs positively correlated with temperature (*i.e.*, higher relative abundance at T > 31°C) were dominated by members of *Pseudoalteromonadaceae*, *Clostridiaceae, Alteromonadaceae* and *Halomonadaceae* and had their highest relative abundance in the HTV sediments collected during the hot season (August; up to 51%). Contrary, the OTUs negatively correlated with temperature (*i.e.*, higher relative abundance at T < 28°C) had their maximum in the LTV and ITV sediments of August (5% each) and in the LTV and HTV of December (15% and 17%, respectively) that experienced temperature between 26.8°C and 27.4°C; such taxa were distributed in 32 phyla, 73 classes and 97 families, with Acidobacteria (8 classes), *Deltaproteobacteria* (mainly *Desulfobacteraceae*), *Gammaproteobacteria* (*Piscirickettsiaceae*), and *Alphaproteobacteria* (unclassified and *Rhodospirillaceae*) as main representants (Supplementary Data S5).

SUPPLEMENTARY TABLES

Supplementary Table S1. Locations, codes, country of origin and GPS coordinates of samples collected along the thermal transect.

Site	Code	Country	Latitude	Longitude	Sampling T (ºC)	MAT (ºC)*
Menai Strait	MS	IS	53°13'32.00"N	4°9'35.00"W	14.0	12.0
Ancona harbor	Anc	IT	43°37'0.00"N	13°50'15.00"E	n.d.	18.3
Bizerte lagoon	Biz	TN	37°10'27.46"N	15°12'7.50"E	19.3	19.35
Mar Chica lagoon	MCh	MA	31°9'31.20"N	29°50'28.20"E	21.3	19.6
Milazo harbor	Mil	IT	38°12'30.10"N	15°15'34.89"E	23.0	20.0
Priolo Gargallo harbor	Pri	IT	37°16'8.90"N	9°53'20.10"E	19.0	20.05
Messina harbor	Mes	IT	38°11'42.27"N	15°34'25.01"E	23.0	20.05
El-Max	ElMax	ET	35°11'57.10"N	2°55'37.60"W	20.0	22.15
Gulf of Aqaba	Aq	HKJ	30°22'0.42"N	25°24'57.00"E	26.5	24.75
KAEC, Thuwal	Ec	SA	22°24'28.02"N	39°7'58.44"E	26.4	28.6[#]
Alkarar lagoon	AL	SA	22°57'29.94"N	38°52'20.88"E	27.9	28.6[#]
Jizan	JM1	SA	16°45'5.22"N	42°2'17.94"E	24.1	29.95
Farasan island	FI/J	SA	16°52'40.02"N	42°10'35.46"E	27.6	29.95[$]
Jizan	JM2	SA	16°45'5.22"N	42°2'17.94"E	30.4	29.95

*Average of annual max sea surface temperatures as calculated by http://www.seatemperature.org/

[#]Data referred to Jeddah

[$]Data referred to Jizan

n.d., not determined

Supplementary Table S2. The linear models describe the effects of temperature (MAT), pH and salinity on the relative optimum temperature of the activity (T_{opt}) tested for six enzyme classes from the total active proteins extracted from sediments. The residual degrees of freedom (d.f.) and R^2 are given. The treatment degrees of freedom and sum of squares only apply to the term added to the model. The Akaike information criterion (AIC) was calculated for each model; a lower AIC (in bold) indicates an improved model.

Enzyme class	Model	Residual d.f.	R^2	AIC
Phosphatase	Intercept	13	–	111.26941
	**Temperature**	**1,12**	**0.81676573**	**89.66354**
	pH	1,12	0.24679656	91.66296
	Salinity	1,12	0.05630128	93.61122
	Temperature+Salinity	2,11	0.81477338	91.64239
	Temperature+pH	2,11	0.81545666	106.17103
	Salinity+pH	2,11	0.81504527	109.30153
	Temperature+Salinity+pH	3,10	0.47789383	112.45814
Transaminase	Intercept	13	–	106.53583
	**Temperature**	**1,12**	**0.7531786**	**93.0106**
	pH	1,12	0.41171606	95.09829
	Salinity	1,12	0.0094856	95.41522
	Temperature+Salinity	2,11	0.6680202	93.52842
	Temperature+pH	2,11	0.70562498	98.86804
	Salinity+pH	2,11	0.70323513	101.10819
	Temperature+Salinity+pH	3,10	0.56543674	108.4024
Beta-galactosidase	Intercept	13	–	112.77638
	**Temperature**	**1,12**	0.7744746	**95.67431**
	pH	1,12	0.16004363	97.46425
	Salinity	1,12	0.08342188	99.01689
	Temperature+Salinity	2,11	0.74827996	97.0361
	Temperature+pH	2,11	0.75619645	109.52674
	Salinity+pH	2,11	0.75586167	112.33471
	Temperature+Salinity+pH	3,10	0.40418889	113.55687
Nuclease	Intercept	13	–	111.90046
	**Temperature**	**1,12**	**0.69382133**	**98.21613**
	pH	1,12	0.26991424	100.2158
	Salinity	1,12	0.04878416	101.81001
	Temperature+Salinity	2,11	0.67382897	100.02237
	Temperature+pH	2,11	0.68314723	106.37621
	Salinity+pH	2,11	0.67830451	109.49616
	Temperature+Salinity+pH	3,10	0.493537	113.20026
Aldo-keto reductases	Intercept	13	–	117.3649
	**Temperature**	**1,12**	**0.75081624**	**103.8547**
	pH	1,12	0.04938345	108.8214
	Salinity	1,12	0.03037934	104.2213
	Temperature+Salinity	2,11	0.59178644	106.3743
	Temperature+pH	2,11	0.74523276	119.3625
	Salinity+pH	2,11	0.65725211	118.6559
	Temperature+Salinity+pH	3,10	0.13327316	118.933
Extradiol dioxygenases	Intercept	13	–	101.90545
	**Temperature**	**1,12**	**0.7810762**	**88.72452**
	pH	1,12	0.2532297	92.20731
	Salinity	1,12	0.162085	89.55571
	Temperature+Salinity	2,11	0.6241025	93.46714
	Temperature+pH	2,11	0.7303676	89.28903
	Salinity+pH	2,11	0.5887075	99.81748
	Temperature+Salinity+pH	3,10	0.6948303	101.42971

Supplementary Table S3. The number of sequences potentially encoding esterases selected from raw sequencing reads of the microbial communities from the investigated samples deposited in the NCBI as reported in the previous studies[2-5]. Sequences were selected using the Blastp algorithm via the DIAMOND v2.0.9 program with default parameters (percentage of identity ≥ 60%; alignment length ≥70; e-value ≤ $1e^{-5}$) against the Lipase Engineering Database (http://www.led.uni-stuttgart.de)[4]. Datasets also included the number of quality-filtered non-redundant proteins identified per each sample analysed by shotgun proteomics.

Location	T (ºC)	Bioproject	NCBI link	N. ORFs	Metagenome		Metaproteome[3,4]	
					N. sequences encoding hydrolases	Rel. ab. (%) candidate hydrolases[2]	N. proteins identified	Rel. ab. (%) proteins
Menai Strait[1]	12.0	PRJNA847771	www.ncbi.nlm.nih.gov/bioproject/PRJNA847771	138782	137	0.10	n.d.	n.d.
Ancona harbour	18.3	PRJNA222664	www.ncbi.nlm.nih.gov/bioproject/?term=PRJNA222664	27893	68	0.24	1149	4.12
Bizerte lagoon	19.35	PRJNA222660	www.ncbi.nlm.nih.gov/bioproject/?term=PRJNA222660	56406	134	0.24	583	1.01
Mar Chica lagoon	19.6	PRJNA222661	www.ncbi.nlm.nih.gov/bioproject/?term=PRJNA222661	44522	99	0.22	324	0.73
Messina harbour	20.05	PRJNA222657	www.ncbi.nlm.nih.gov/bioproject/?term=PRJNA222657	40077	63	0.16	794	1.98
Priolo Gargallo harbour	20.05	PRJNA222658	www.ncbi.nlm.nih.gov/bioproject/?term=PRJNA222658	5858	11	0.19	59	1.01
El-Max	22.15	PRJNA222666	www.ncbi.nlm.nih.gov/bioproject/?term=PRJNA222666	64758	165	0.25	452	0.70
Gulf of Aqaba	24.75	PRJNA222667	www.ncbi.nlm.nih.gov/bioproject/?term=PRJNA222667	52322	154	0.29	557	1.06

[1]From enrichment sample incubated at 20ºC.
[2]Relative abundance of sequences encoding presumptive target hydrolases, referred to as the total number of Open Reading Frames (ORF) mapped in the relative metagenomes.
[3]Raw proteomic information in Supplementary Data S2.
[4]Relative abundance of proteins identified in the proteomes, referred to as the total number of ORF mapped in the relative metagenomes.

Supplementary Table S4. Locations, sediment depths (m), mean annual temperature (MAT, °C) and annual temperature variability (TV, °C) of the nice stations; HTV, high temperature variability; ITV, intermediate temperature variability; LTV, low temperature variability. Measurements recorded were analysed and divided into three categories: T ≤ 27°C, 27°C < T < 31°C, and T ≥ 31°C. Fractions of measures belonging to one of the three categories are reported as a percentage (%T).

Station	Coordinates	Depth (m)	MAT (°C)	TV (°C)	%T ≤27°C	%T >27°C/>31°C	%T ≥31°C
HTV-1	22°16'58.99"N, 39°5'8.30"E	3.7	29.45	12.84			
HTV-2	22°16'46.24"N, 39°5'7.57"E	2.6	29.48	12.78	41.45	13.41	45.14
HTV-3	22°16'34.44"N, 39°5'3.39"E	3.7	29.46	12.81			
ITV-1	22°18'00.82"N, 39°3'20.59"E	25.3	28.52	8.10			
ITV-2	22°17'45.06"N, 39°3'9.70"E	25.5	28.52	8.80	45.04	42.88	12.08
ITV-3	22°17'36.54"N, 39°2'52.40"E	25.6	28.51	8.85			
LTV-1	22°19'40.23"N, 39°1'34.65"E	54.4	26.95	6.74			
LTV-2	22°18'48.10"N, 39°1'16.95"E	52.0	26.94	6.72	86.64	13.36	0.00
LTV-3	22°18'23.69"N, 39° 1'7.93"E	51.8	26.95	6.72			

Supplementary Table S5. *In-situ* temperatures, pH, salinity and light (PAR) are reported for both sediments and the 10 cm water-layer covering the sediments in the two sampling periods of August and December 2016.

Station	Sediment T (°C)		Water T (°C)		pH		Salinity (g/l)		Light (PAR)
	Aug	Dec	Aug	Dec	Aug	Dec	Aug	Dec	
HTV-1	32.8	26.7	33.4	26.8	7.96	7.82	40	38	69.1
HTV-2	33.1	26.9	33.4	27.3	8.12	7.73	40	38	69.1
HTV-3	33.2	26.8	33.3	26.9	7.9	7.88	39	38	69.1
ITV-1	30.7	27.5	31.4	27.9	8.02	7.82	40	38	69.1
ITV-2	30.6	27.7	31.6	28.0	8.12	7.79	39	39	69.1
ITV-3	30.5	28.1	31.5	28.2	7.82	7.79	40	38	69.1
LTV-1	27.1	26.2	28.5	27.4	7.69	8.02	38	39	55.3
LTV-2	27.4	26.5	28.5	27.7	7.87	7.89	38	38	55.3
LTV-3	27	26.6	28.5	27.9	7.68	7.91	39	36	55.3

Supplementary Table S6. (**a**) Multiple comparison two-sided *t*-test of bacterial communities associated with HTV, ITV and LTV sediments; FDR correction was applied, *t* and *p*-values are reported. (**b**) Cross-validation by the canonical analysis of principal coordinates of the sediment bacterial communities' classification in the three thermal variations (HTV, ITV and LTV). Sediments collected in the two seasons (August and December) were analysed. Permutation test, first squared canonical correlation: delta_1^2 = 0.99506, $p = 0.001$.

**(a) Thermal variability**	***t***	***P***
LTV vs ITV	2.4656	0.001
LTV vs HTV	2.8711	0.001
ITV vs HTV	2.0035	0.002

**(b) Season**	**Class**	**HTV**	**ITV**	**LTV**	**Total**	**% correct**
August	HTV	9	0	0	9	100
	ITV	0	9	0	9	100
	LTV	0	0	9	9	100
December	HTV	9	0	0	9	100
	ITV	0	9	0	9	100
	LTV	0	0	9	9	100

Supplementary Table S7. (**a**) Ordination one-way ANOVA to evaluate the differential distribution of each genus across the cultivable communities obtained from the sediments HTV, ITV, and LTV and incubated at 20°C, 30°C and 40°C. Numbers indicate the ANOVA *p*-values resulting from comparisons of the relative abundance (average of replicates, $n = 9$) within each genus across the three sediments at each temperature tested. (**b**) Permutational MANOVA comparison among the three different TVs within each temperature tested with a two-sided *t*-test; FDR correction for multiple comparisons was applied.

**(a) Genus**	**August, HTV vs. ITV vs. LTV**			**December, HTV vs. ITV vs. LTV**		
	**20°C**	**30°C**	**40°C**	**20°C**	**30°C**	**40°C**
Vibrio	0.231	0.118	0.918	0.185	0.337	0.0743
Bacillus	0.105	0.473	0.806	0.261	0.357	0.091
Photobacterium	0.303	0.246	0.054	0.151	0.351	0.423
Enterovibrio	0.579	0.5523	0.396	0.413	0.856	0.235
Ferrimonas	0.124	0.485	0.108	0.398	0.921	0.455
Virgibacillus	0.357	0.331	0.659	0.846	0.081	0.061
Hypnocyclicus	0.592	0.724	0.485	0.523	0.312	0.401
Marinobacter	0.3654	0.382	0.612	0.335	0.346	0.388
Paenibacillus	0.382	0.665	0.894	0.537	0.542	0.373
Others (<0.5%)	0.737	0.188	0.439	0.229	0.614	0.137
Unc Bacteria	0.179	0.517	0.598	0.709	0.267	0.0935

**(b) Temperature**	**PERMANOVA *p*-value**	
	**August**	**December**
HTV vs. ITV vs. LTV at 20°C	0.975	0.325
HTV vs. ITV vs. LTV at 30°C	0.492	0.199
HTV vs. ITV vs. LTV at 40°C	0.095	0.092

Supplementary Table S8. Amount of proteins extracted from 100 ± 5 g of sediment and expressed as the total amount extracted from the three biological replicates (values of mean ± SD calculated using Microsoft Excel 2019) for each thermal environment station in both August and December sampling.

Station	Protein amount [mg]	
	August	December
HTV-1	2.18 ± 0.32	3.31 ± 0.12
HTV-2	9.53 ± 1.83	4.81 ± 0.30
HTV-3	2.06 ± 0.28	4.25 ± 0.08
ITV-1	3.99 ± 0.05	5.60 ± 0.03
ITV-2	4.39 ± 0.12	7.97 ± 0.58
ITV-3	8.17 ± 0.55	9.01 ± 0.26
LTV-1	6.19 ± 0.26	5.64 ± 0.75
LTV-2	3.15 ± 0.02	4.33 ± 0.17
LTV-3	7.38 ± 0.43	8.63 ± 0.06

Supplementary Table S9. Number of sequences, number of OTUs (richness) and Good's coverage values were reported for sediments collected in August and December.

August	N. seq.	N. OTUs	Good's cov.	December	N. seq.	N. OTUs	Good's cov.
LTV-1-1	21536	3288	0.93	LTV-1-1	35272	4422	0.96
LTV-1-2	12508	2315	0.90	LTV-1-2	138757	7367	0.99
LTV-1-3	21487	3253	0.93	LTV-1-3	99042	6432	0.99
LTV-2-1	28719	3355	0.95	LTV-2-1	124562	6653	0.99
LTV-2-2	20585	2411	0.95	LTV-2-2	136504	5512	0.99
LTV-2-3	35059	2979	0.97	LTV-2-3	144818	5771	1.00
LTV-3-1	33863	1907	0.97	LTV-3-1	113595	6128	0.99
LTV-3-2	18809	2445	0.94	LTV-3-2	121300	6776	0.99
LTV-3-3	13388	2135	0.92	LTV-3-3	173996	6659	1.00
ITV-1-1	7289	1086	0.92	ITV-1-1	62556	5404	0.97
ITV-1-2	31312	2952	0.96	ITV-1-2	49390	4834	0.96
ITV-1-3	15988	2025	0.93	ITV-1-3	80666	5295	0.98
ITV-2-1	18549	2201	0.94	ITV-2-1	106564	5912	0.99
ITV-2-2	24807	2804	0.95	ITV-2-2	135785	6488	0.99
ITV-2-3	7977	1435	0.90	ITV-2-3	133627	5523	0.99
ITV-3-1	29684	3516	0.95	ITV-3-1	148165	5748	0.99
ITV-3-2	14886	1706	0.94	ITV-3-2	122367	5091	0.99
ITV-3-3	32454	3036	0.96	ITV-3-3	96991	4947	0.99
HTV-1-1	44363	2596	0.97	HTV-1-1	58141	5058	0.97
HTV-1-2	5501	734	0.93	HTV-1-2	63154	5252	0.97
HTV-1-3	3507	135	0.98	HTV-1-3	47626	4645	0.97
HTV-2-1	24696	2069	0.95	HTV-2-1	53984	4461	0.97
HTV-2-2	38078	1509	0.98	HTV-2-2	81563	5558	0.98
HTV-2-3	83613	2496	0.99	HTV-2-3	100313	5649	0.99
HTV-3-1	61931	4002	0.98	HTV-3-1	97131	4395	0.99
HTV-3-2	39351	2799	0.97	HTV-3-2	159267	5442	0.99
HTV-3-3	31298	2954	0.96	HTV-3-3	137998	6109	0.99

Supplementary Table S10. Number of sequences, number of OTUs (richness) and Good's coverage values were reported for the cultivable bacterial communities obtained from LTV, ITV and HTV sediments collected in August and December after cultivation at 20°C, 30°C, and 40°C in the laboratory (T°C*).

August	T°C*	N. OTUs	N. seqs	Good's	December	T°C*	N. OTUs	N. seqs	Good's
LTV	20	52	141487	99.99	LTV	20	77	97092	99.99
LTV	30	56	144049	100.00	LTV	30	82	82633	99.99
LTV	40	87	133601	100.00	LTV	40	62	99418	99.99
LTV	20	45	46339	99.97	LTV	20	61	105412	99.99
LTV	30	56	149151	99.99	LTV	30	83	121520	99.99
LTV	40	50	183866	100.00	LTV	40	81	94686	99.99
LTV	20	58	181186	100.00	LTV	20	65	84165	99.98
LTV	30	97	146319	100.00	LTV	30	66	105953	99.98
LTV	40	71	147299	99.99	LTV	40	47	96036	99.99
LTV	20	50	57373	99.98	LTV	20	82	94298	99.99
LTV	30	79	95110	99.99	LTV	30	90	110968	99.98
LTV	40	67	108084	99.98	LTV	40	50	117064	99.99
LTV	20	58	155486	99.99	LTV	20	65	105715	99.98
LTV	30	73	138761	99.99	LTV	30	60	126791	99.99
LTV	40	44	118114	99.99	LTV	40	26	103291	99.99
LTV	20	63	18976	99.95	LTV	20	62	14060	99.91
LTV	30	63	154035	100.00	LTV	30	80	136138	99.99
LTV	40	43	122338	99.99	LTV	40	37	94410	99.99
LTV	20	54	79157	99.97	LTV	20	48	94415	99.98
LTV	30	49	96907	99.99	LTV	30	46	66991	99.98
LTV	40	33	64243	99.99	LTV	40	45	127426	99.99
LTV	20	52	81792	99.98	LTV	20	78	97276	99.99
LTV	30	75	65678	99.99	LTV	30	59	81820	99.99
LTV	40	44	60667	99.98	LTV	40	41	52274	99.98
LTV	20	55	65146	99.98	LTV	20	50	61568	99.98
LTV	30	67	100711	99.99	LTV	30	76	73835	100.00
LTV	40	39	78640	99.99	LTV	40	44	68763	99.99
ITV	20	45	64904	99.99	ITV	20	69	100602	99.98
ITV	30	69	110019	99.99	ITV	30	73	77307	99.99
ITV	40	53	125229	99.99	ITV	40	38	83710	99.99
ITV	20	49	202862	99.99	ITV	20	55	84979	99.99
ITV	30	76	160059	99.99	ITV	30	58	132152	99.99
ITV	40	48	158266	99.99	ITV	40	28	99701	99.99
ITV	20	56	133197	99.99	ITV	20	55	134242	99.99
ITV	30	70	142734	99.99	ITV	30	47	92911	99.99
ITV	40	53	119098	99.99	ITV	40	24	98616	99.98
ITV	20	63	125795	99.99	ITV	20	53	125759	99.99
ITV	30	68	118781	99.99	ITV	30	75	88195	99.98
ITV	40	63	87442	99.99	ITV	40	43	86757	99.99
ITV	20	60	132480	99.99	ITV	20	72	84351	99.99
ITV	30	64	156308	99.99	ITV	30	78	84823	99.99
ITV	40	67	98875	99.98	ITV	40	29	85496	99.98
ITV	20	65	130196	99.99	ITV	20	59	85474	99.99
ITV	30	67	115945	100.00	ITV	30	49	83428	99.98
ITV	40	58	143764	99.99	ITV	40	63	95078	99.99
ITV	20	68	77174	99.98	ITV	20	76	97175	99.99
ITV	30	60	88381	99.99	ITV	30	96	70869	99.98
ITV	40	20	58749	99.97	ITV	40	68	72637	99.99
ITV	20	57	87601	99.99	ITV	20	43	65248	99.98
ITV	30	51	110279	99.99	ITV	30	72	66655	99.99
ITV	40	41	100462	99.99	ITV	40	44	60676	99.98
ITV	20	52	47943	99.99	ITV	20	66	75593	99.98
ITV	30	81	74709	99.98	ITV	30	66	148643	99.99
ITV	40	71	125421	99.99	ITV	40	51	44580	99.98

HTV	20	72	192440	100.00	HTV	20	68	104844	99.99
HTV	30	39	155298	99.99	HTV	30	67	89805	99.99
HTV	40	60	147380	99.99	HTV	40	56	103633	99.99
HTV	20	74	101525	99.99	HTV	20	46	88389	99.99
HTV	30	74	109812	99.99	HTV	30	58	79930	99.99
HTV	40	58	201382	100.00	HTV	40	52	87105	99.99
HTV	20	64	161847	99.99	HTV	20	55	112584	99.99
HTV	30	78	99503	99.99	HTV	30	59	105267	99.99
HTV	40	41	160450	100.00	HTV	40	67	109	91.74
HTV	20	38	103930	99.99	HTV	20	76	70741	99.99
HTV	30	44	128081	100.00	HTV	30	87	86208	99.98
HTV	40	48	123966	99.99	HTV	40	76	66044	99.98
HTV	20	68	145556	99.99	HTV	20	63	85083	99.98
HTV	30	70	134118	99.99	HTV	30	74	100658	99.99
HTV	40	60	129287	100.00	HTV	40	70	90757	99.99
HTV	20	58	58805	99.99	HTV	20	62	78740	99.99
HTV	30	59	127614	99.99	HTV	30	79	122314	99.99
HTV	40	37	154247	99.99	HTV	40	63	109981	99.99
HTV	20	67	97313	99.99	HTV	20	77	102905	99.99
HTV	30	44	106017	99.99	HTV	30	77	82556	99.99
HTV	40	68	118668	99.99	HTV	40	66	90285	99.98
HTV	20	53	38814	99.98	HTV	20	63	91616	99.98
HTV	30	64	100016	100.00	HTV	30	71	115295	99.99
HTV	40	31	131176	99.99	HTV	40	72	115984	99.99
HTV	20	62	150423	100.00	HTV	20	19	100110	99.99
HTV	30	64	96831	99.99	HTV	30	88	97108	99.98
HTV	40	41	98039	99.99	HTV	40	117	215923	100.00

SUPPLEMENTARY FIGURES

Supplementary Figure S1. Schematic representation of the pipeline implemented in this work to investigate enzyme activities in microbial communities associated with an ample set of marine samples. Shown are the source of enzymes (either present in native proteomes or single enzymes produced in *E. coli*) and the methods employed. The figure includes icons created with BioRender.com.

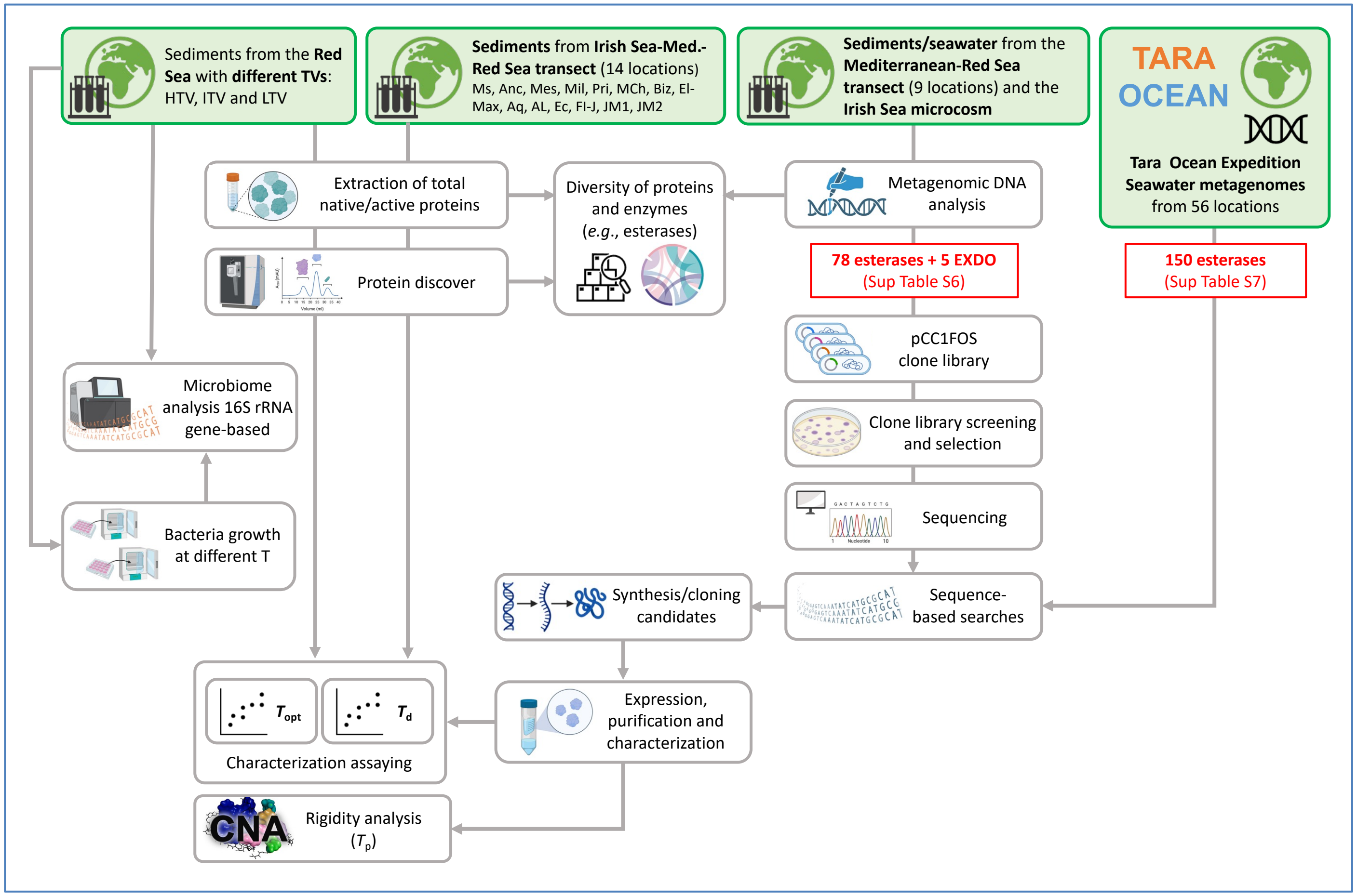

Supplementary Figure S2. Sypro-stained two-dimensional SDS-PAGE (2-DE, 12% acrylamide) of the native/active proteins extracted from sediment samples herein investigated. The number of spots, which may be underestimated given the complexity of the gels, is quantified using ImageJ (version 1.54b) program. For each location is reported the number of spots counted per gel, followed by values of mean of triplicate measurements using ImageJ ± SD calculated using Microsoft Excel 2019: Alkarar lagoon (AL, 1: 547; 2: 547; 3: 554; mean: 549±4); Ancona harbor (Anc, 1: 836; 2: 846; 3: 854; mean: 845±9); Gulf of Aqaba (Aq, 1: 554; 2: 554; 3: 566; mean: 558±7); Jizan (JM1, 1: 523; 2: 603; 3: 523; mean: 550±46); Jizan (JM2, 1: 582; 2: 406; 3: 595; mean: 528±105); Bizerte lagoon (Biz, 1: 551; 2: 573; 3: 560; mean: 561±11); Economic City (EC, 1: 631; 2: 466; 3: 631; mean: 576±95); El-Max (ElMax, 1: 560; 2: 574; 3: 465; mean: 530±64); Farasan island (FI/J, 1: 821; 2: 821; 3: 821; mean: 821±0); Mar Chica lagoon (MCh, 1: 555; 2: 558; 3: 555; mean: 556±2); Messina harbour (Mes, 1: 808; 2: 809; 3: 821; mean: 813±7); Milazo harbour (Mil, 1: 920; 2: 921; 3: 986; mean: 942±37); Menai Strait (MS, 1: 765; 2: 748; 3: 785; mean: 766±18). The 2-DE was performed using 11 cm IPG strips in the pH range of 3–10 and molecular weight markers ranging from 10 to 250 kDa. The chaperonins Cpn60 from *Oleispira antarctica* RB-8 (GenBank: CAD43724.1)[6] is used as a reference protein (see red star in the gels). The 2-DE was performed by using a validated pooling strategy[7], in which proteins extracted from three independent biological (sediments) replicates were mixed in equal amount to conform a pool and 150 µg of protein mix is loaded in the gel. For each site, the total amount of proteins extracted from biological triplicates of 100 g sediments is as follows: MS, 290±7, Anc, 452±5 µg; Mil, 314±23 µg, Mes, 389±27 µg, Biz, 296±45 µg; MCh, 283±12 µg; ElMax, 349±16 µg; Aq, 362±27 µg; AL, 409±22 µg; EC, 378±53 µg; FI/J, 915±23 µg; JM2, 267±31 µg; JM1, 805±17 µg (values of mean ± SD calculated using Microsoft Excel 2019). All uncropped scan gels are provided as Source data files.

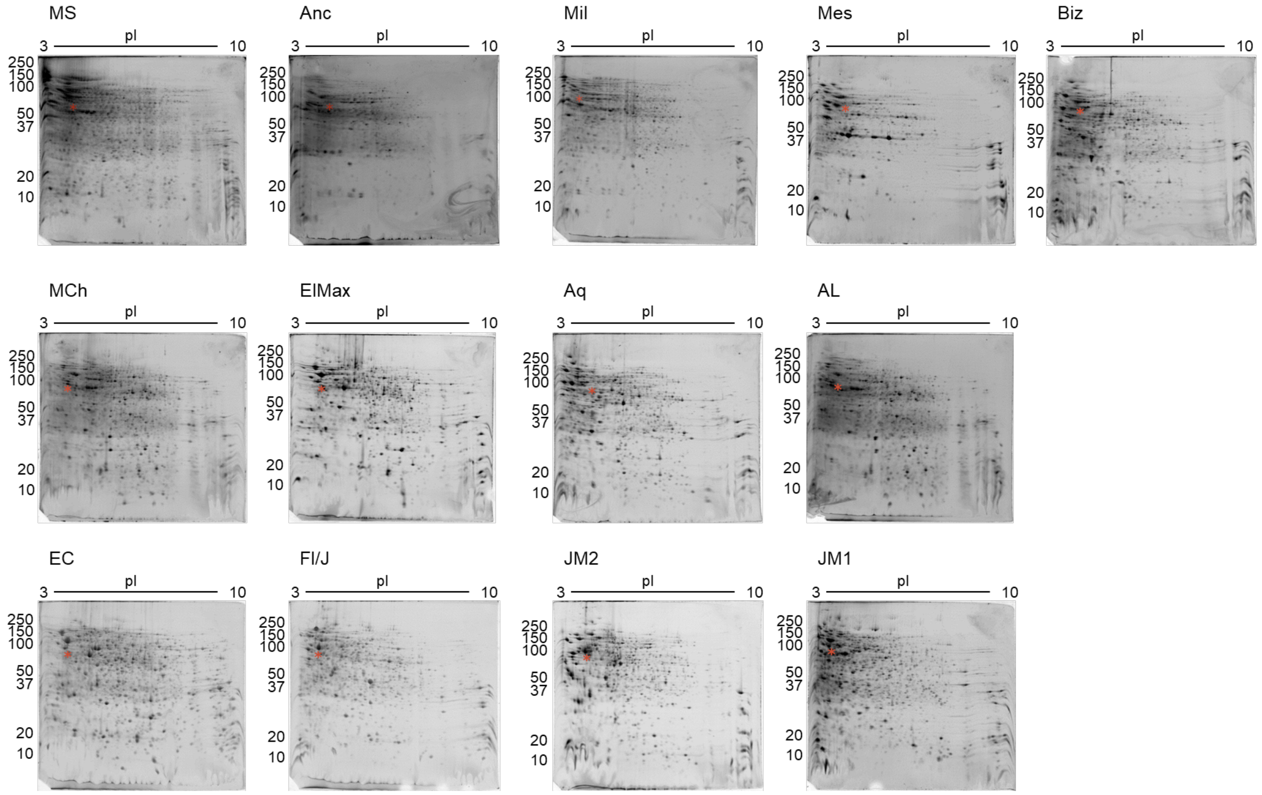

Supplementary Figure S3. Venn Diagram representing the number of sequences potentially encoding esterases from raw sequencing reads of the microbial communities from the eight samples from which both the proteomes and the raw metagenome sequences were available (see Supplementary Table S3). Sequences were selected by using DIAMOND-BLASTP search tool with default parameters. As shown, any of sequences encoding esterases (947 in total) are shared among all samples.

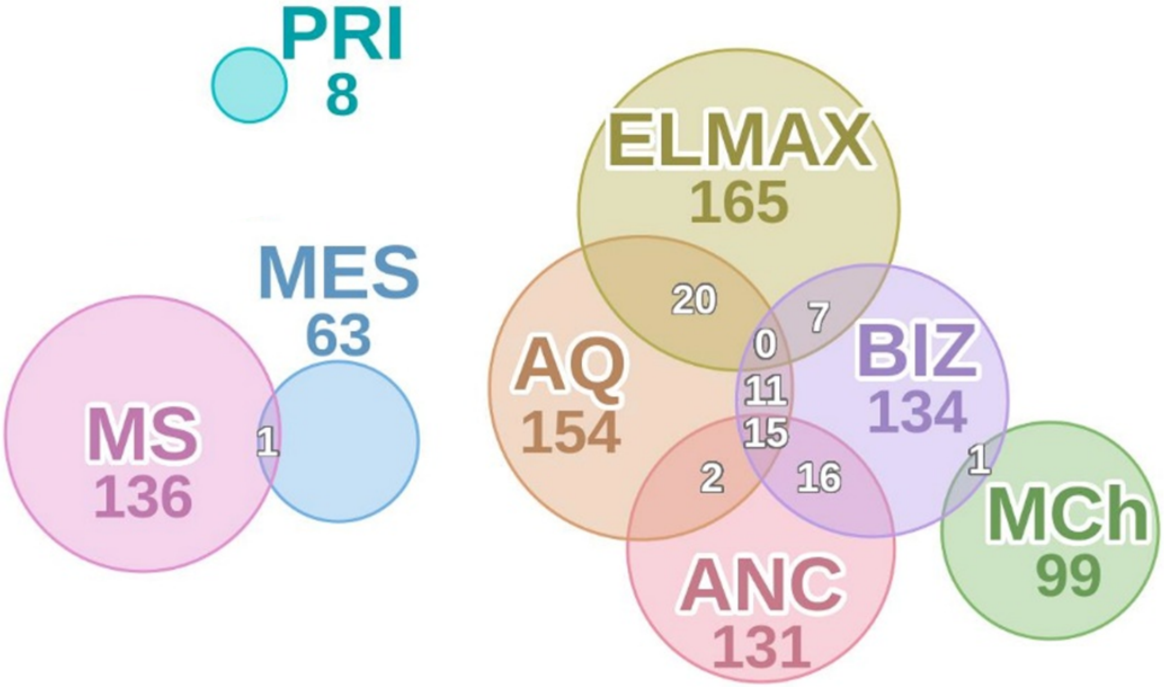

Supplementary Figure S4. Thermal characteristics (T_d, T_{opt}, T_p) of 78 individual enzymes of sediment microbial communities from 14 marine locations across a broad latitudinal gradient (from the Irish Sea to the Red Sea) compared to salinity (**a-c**) and pH (**d-f**) at the sampling site from which each of the enzymes was selected (salinity and pH retrieved from Bio-ORACLE, see Supplementary Data S3). For enzymatic activity determination, see details in Methods. T_{opt} and T_d are plotted as mean (n = 3) and SD are reported is Supplementary Data S3; T_p are plotted as mean (n = 5) and SEM are reported in Supplementary Data S3. Linear regressions (blue lines) are computed by the function lm() in R and the 95% confidence intervals are shown as blue areas. (**a**) R^2=0.05054, $F_{1,76}$=4.045, p=0.05784; (**b**) R^2=0.04088, $F_{1,76}$=3.239, p=0.0759; (**c**) R^2=0.03614, $F_{1,60}$=2.249, p=0.1389; (**d**) R^2=0.08973, $F_{1,76}$=2.492, p=0.067713; (**e**) R^2=0.1981, $F_{1,76}$=18.77, p=0.0048; (**f**) R^2=0.07213, $F_{1,60}$=4.664, p=0.0348.

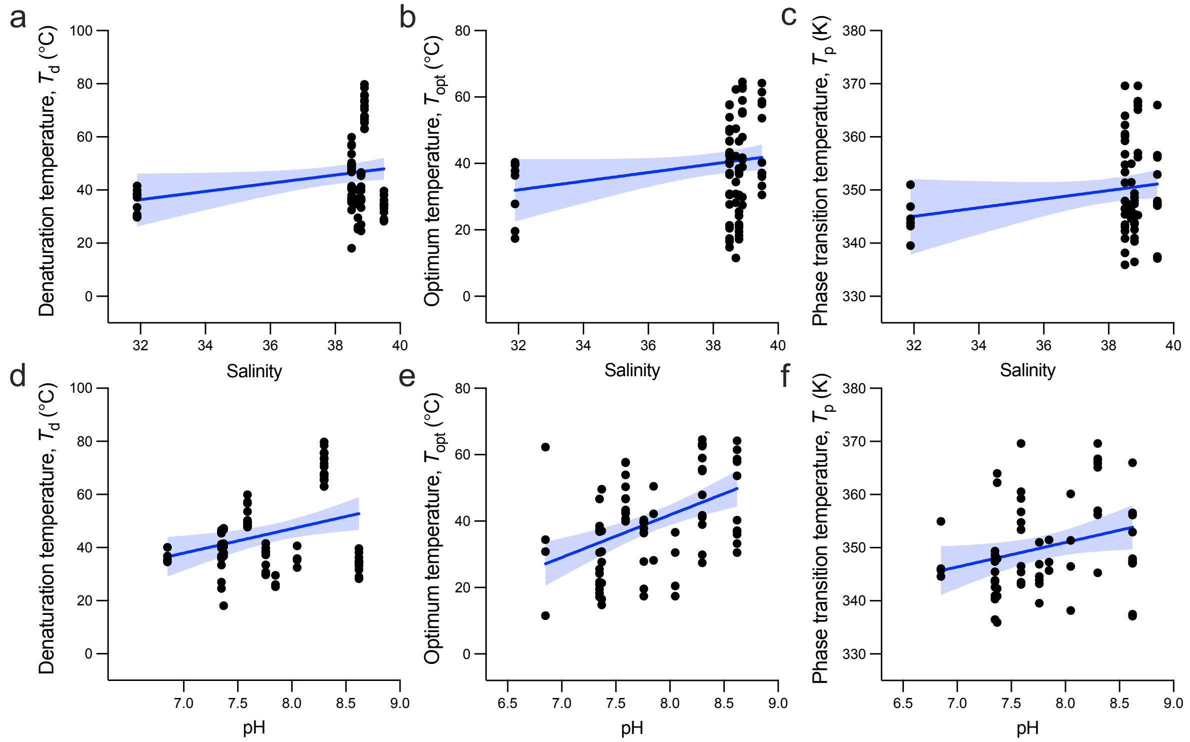

Supplementary Figure S5. (**a**) Denaturation temperature (T_d) and (**b**) optimal temperature (T_{opt}) patterns as a function of the MAT of five EXDOs individual enzymes from sediment microbial communities. Data are plotted as mean (*n* = 3) and SD are reported in Supplementary Data S3. Linear regressions (blue lines) were calculated by the function lm() in R and the 95% confidence intervala are shown as blue areas. In each graph it is reported the *p*-value and the R^2; (**a**) $F_{1,3}$=89.09 and (**b**) $F_{1,3}$=16.66.

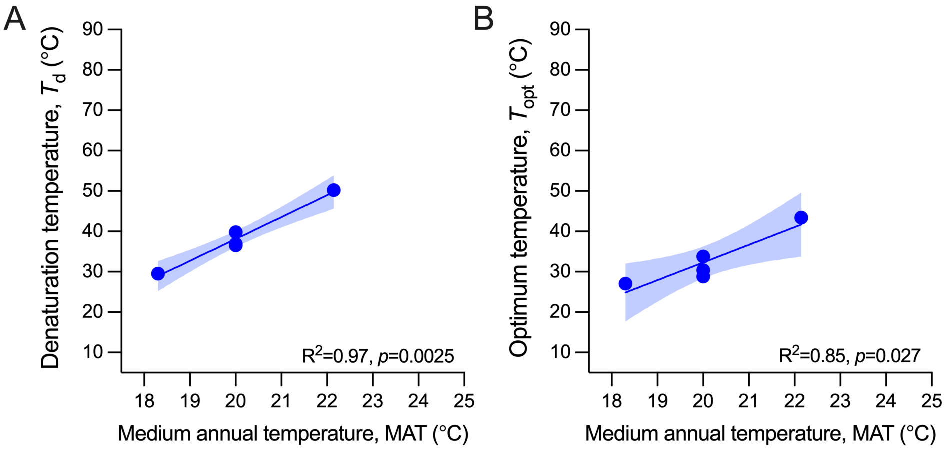

Supplementary Figure S6. Map with locations of all 56 Tara Ocean sites. Triangles represent the sites with a colour-codes of site temperature. The circles inside the triangles represent the denaturing temperature of the enzymes (colour-coded). The map was made with QGIS v3.10[8] employing a physical worldwide base map from Natural Earth[9].

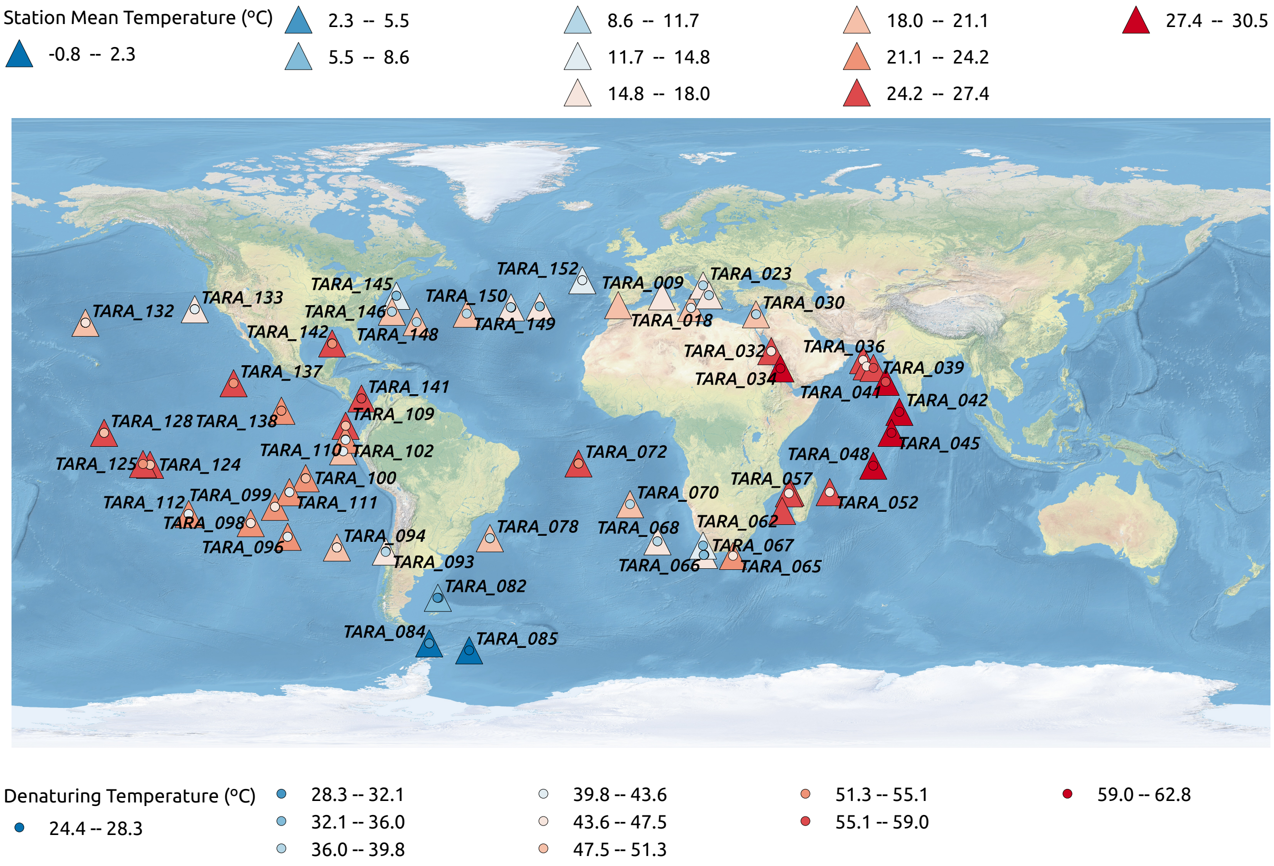

Supplementary Figure S7. Thermal characteristics (T_d, T_{opt}, T_p) of 150 esterases synthesized, expressed, and purified from Tara Ocean metagenomes compared to salinity (**a-c**) and pH (**d-f**) at the sampling site from which each of the enzymes were selected. Salinity and pH were obtained from Bio-ORACLE (Supplementary Data S4) For enzymatic activity determination, see details in Methods. T_{opt} and T_d are plotted as mean (n = 3) and SD are reported is Supplementary Data S4; Tp are plotted as mean (n = 5) and SEM are reported in Supplementary Data S4. Linear regressions (blue lines) are computed by the function lm() in R and the 95% confidence intervals are shown as blue areas. (**a**) R^2=0.01742, $F_{1,147}$=2.606, p: 0.1086; (**b**) R^2=5.2E-006, $F_{1,147}$=0.0007702, p=0.9779; (**c**) R^2=0.0001188, $F_{1,141}$=0.01675, p=0.8972; (**d**) R^2=0.0002313, $F_{1,50}$=0.01157, p=0.9148; (**e**) R^2=0.0008906, $F_{1,50}$=0.04457, p=0.8337; (**f**) R^2=0.01232, $F_{1,45}$=0.5611, p=0.4577.

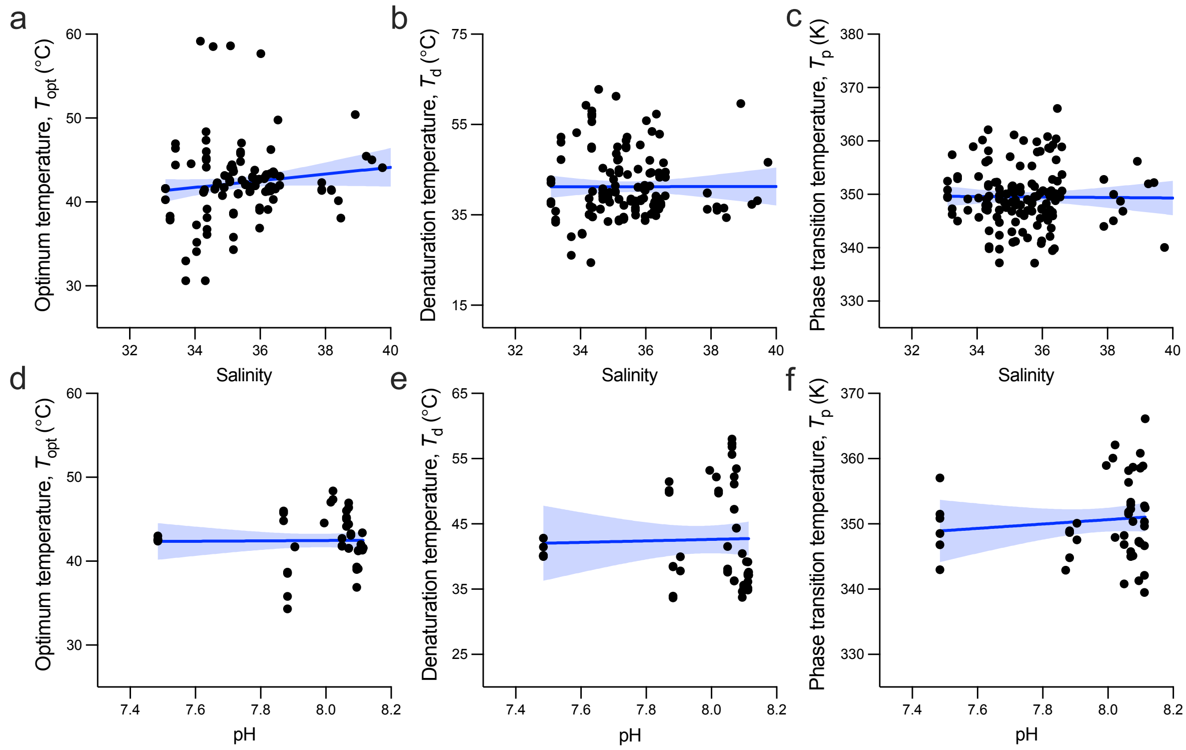

Supplementary Figure S8. The enzymes MAS (Multiple Sequence Alignment) was done using ClustalW[10], as it is implemented in MEGA11[11]. Employing the MAS obtained, the Maximum Likelihood (ML) phylogenetic tree was done using the WAG+F+G substitution model, letting the remaining parameters as default, using MEGA11[11]. The WAG+F+F model was selected because it had the lowest BIC (Bayesian Information Criterion) and AIC (Akaike Information Criterion) among all other models tested in MEGA11[11]. The construction of the unrooted circular tree figure was performed in R[12] using ggtree[13-15] and ggtreeExtra[16] packages. The MAT of the site and family of enzymes are indicated in the tree. Abbreviations as follows: F, Family; CE, Carbohydrate Esterase; MCPH, Meta-Cleavage Product Hydrolase.

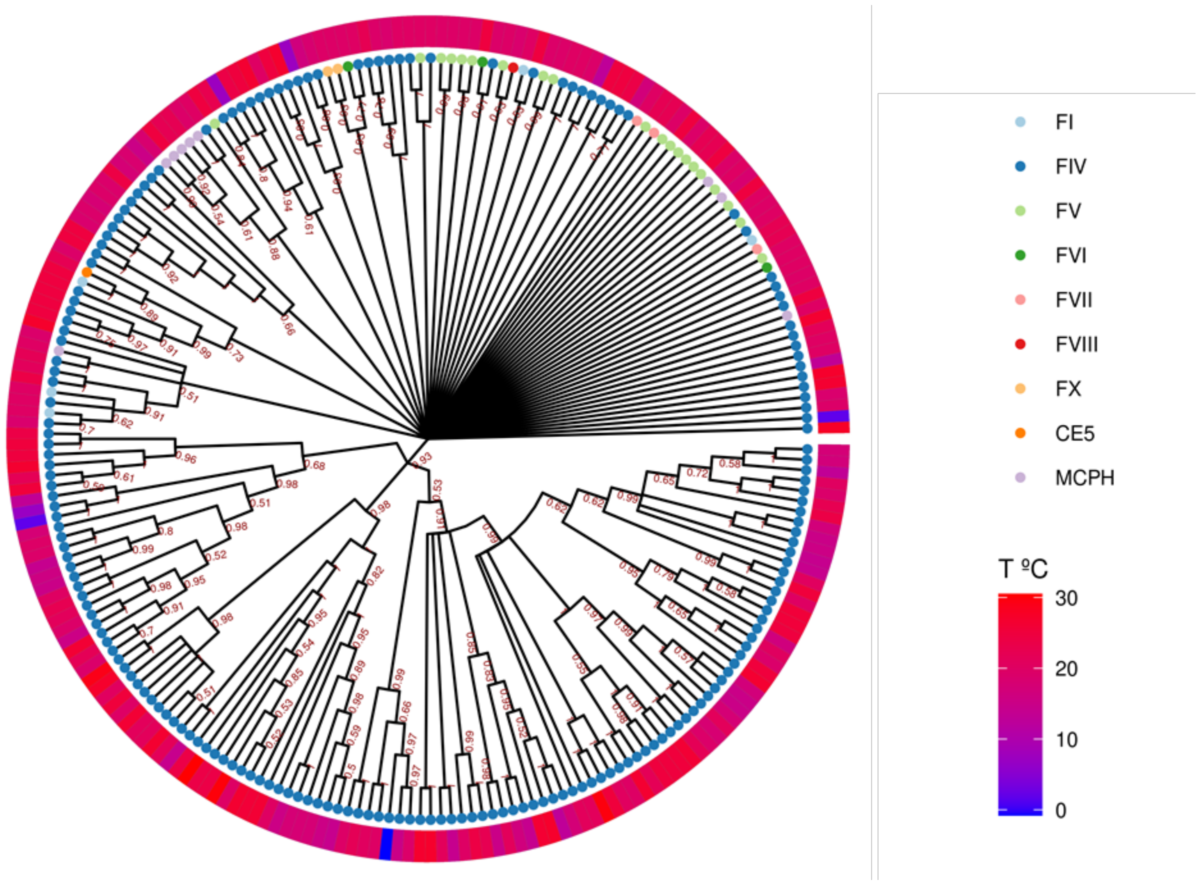

Supplementary Figure S9. Coomassie-stained SDS-PAGE (12% acrylamide) of the protein extracts from the sediments of the three thermal variabilities HTV, ITV and LTV collected in August and December. The SDS-PAGE was performed by using a validated pooling strategy[7], in which proteins extracted from three independent biological (sediments) replicates were mixed in equal amounts to conform a pool, and 10 µg of protein mix was loaded in the gel. The original gel is provided as Source Data.

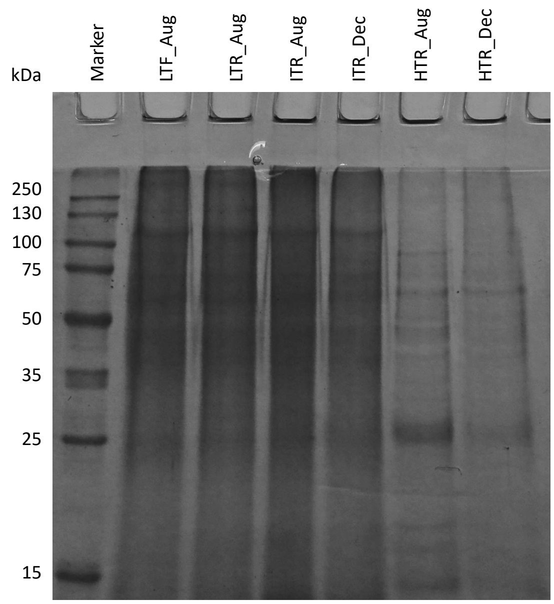

Supplementary Figure S10. (**a**) Temperature decay relationships of the microbial communities' similarities (Bray-Curtis) among the sampled sediments. (**b**) Linear relationship between richness of sediments' bacterial communities (black line)/evenness (grey line) and sampling temperature. Line equation and R-squared values are shown in the graph.

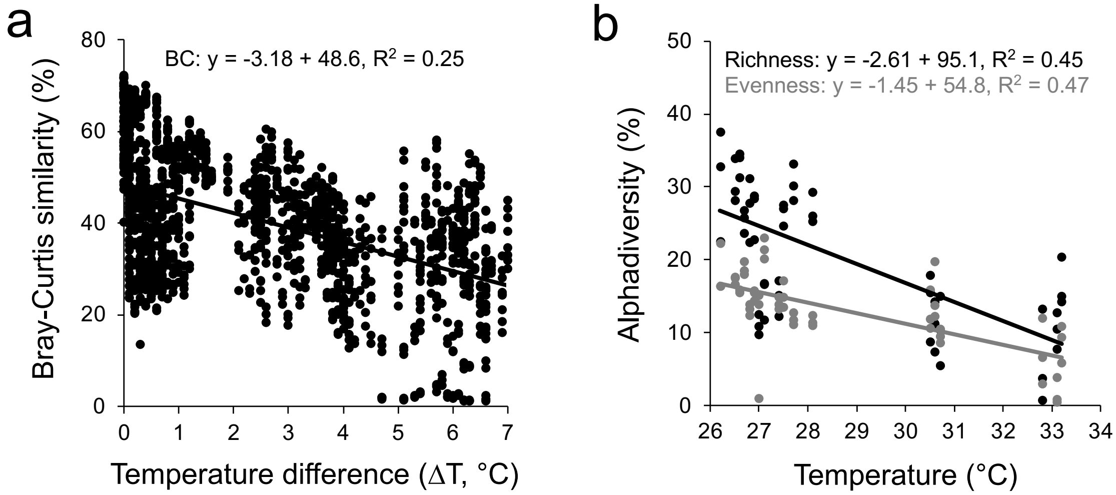

Supplementary Figure S11. Values of OD_{600} are reported as mean ± standard error (n = 3 biological replicates) for bacterial culture obtained from HTV, ITV and LTV sediments and incubated at 10°C, 20°C, 30°C, 40°C, 50°C and 60°C.

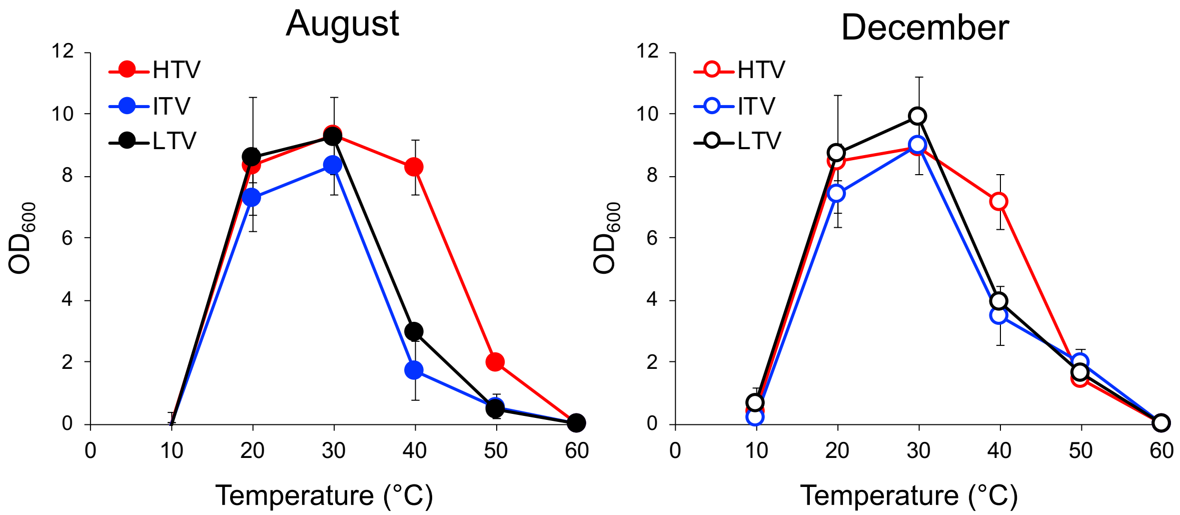

SUPPLEMENTARY REFERENCES

1. Hitch, T.C.A. and Clavel, T. A proposed update for the classification and description of bacterial lipolytic enzymes. *PeerJ* **2019**, e7249 (2019).
2. Bauer, T.L. *et al*. The modular structure of α/β-hydrolases. *FEBS J.* **287**, 1035–1053 (2020).
3. Bargiela, R. *et al.* Bacterial population and biodegradation potential in chronically crude oil-contaminated marine sediments are strongly linked to temperature. *Sci. Rep.* **5**, 11651 (2015).
4. Bargiela, R. *et al.* Degradation network reconstruction in uric acid and ammonium amendments in oil-degrading marine microcosms guided by metagenomic data. *Front. Microbiol.* **6**, 1–12 (2015).
5. Gertler, C. *et al.* Conversion of uric acid into ammonium in oil-degrading marine microbial communities: a possible role of halomonads. *Microb. Ecol.* **70**, 724–740 (2015).
6. Kube, M. *et al*. Genome sequence and functional genomic analysis of the oil-degrading bacterium *Oleispira antarctica*. *Nat. Comm.* **4**, 2156 (2013).
7. uiz-Ruiz S, Sanchez-Carrillo S, Ciordia S, Mena MC, Méndez-García C, Rojo D, Bargiela R, Zubeldia-Varela E, Martínez-Martínez M, Barbas C, Ferrer M, Moya A. Functional microbiome deficits associated with ageing: Chronological age threshold. Aging Cell. 2020 Jan;19(1):e13063.
8. QGIS Development Team (QGIS Association), 2019. QGIS Geographic Information System v3.10 (A Coruña). https://www.qgis.org/en/site/.
9. Natural Earth. Free vector and raster map data. https://www.naturalearthdata.com.
10. Thompson, *et al*. CLUSTAL W: improving the sensitivity of progressive multiple sequence alignment through sequence weighting, position-specific gap penalties and weight matrix choice. *Nucleic Acids Res.*, **22**, 4673–4680 (1994).
11. Koichiro *et al*. MEGA11: Molecular Evolutionary Genetics Analysis Version 11, *Mol. Biol. Evol.* **38**, 3022–3027 (2021).
12. R Core Team. R: A language and environment for statistical computing. R Foundation for Statistical Computing, Vienna, Austria (2022).
13. Guangchuang. Using ggtree to visualize data on tree-like structures. *Curr. Protoc. Bioinform.* **69**, e96 (2020).
14. Guangchuang *et al*. Two methods for mapping and visualizing associated data on phylogeny using ggtree. *Mol. Biol. Evol.* **35**, 3041-3043 (2018).
15. Guangchuang *et al*. ggtree: an R package for visualization and annotation of phylogenetic trees with their covariates and other associated data. *Methods Ecol. Evol.* **8**, 28-36 (2017).
16. Xu *et al*. ggtreeExtra: Compact visualization of richly annotated phylogenetic data. *Mol. Biol. Evol.* **38**, 4039-4042 (2021).
