# Supplementary material for: Enzyme adaptation to habitat thermal legacy shapes the thermal plasticity of marine microbiomes
Source: Nat Commun. 2023 Feb 24;14:1045. doi: 10.1038/s41467-023-36610-0 (PMC9958047; doi:10.1038/s41467-023-36610-0)
Supplement: Supplementary file 2 — Description of Additional Supplementary Files [file 41467_2023_36610_MOESM2_ESM.pdf]

**Description of Additional Supplementary Files**

File Name: **Supplementary Data S1**
Description: Enzymatic activity for esterase, extradiol dioxygenases, phosphatases, beta-galactosidases, nucleases, transaminases and aldo-keto reductases of total microbial proteins extracted from microbial communities inhabiting marine sediments from Iris Sea, Mediterranean and Red Sea (Supplementary Table S1). Values are reported as absorbance per minute (average and standard deviation, $n = 3$).

File Name: **Supplementary Data S2**
Description: Quality-filtered non-redundant proteins identified from seven of the sediment samples herein investigated, Ancona harbour (ANC), Priolo Gargallo harbour (PRI), Messina harbour (MES), Gulf of Aqaba (AQ), El-Max (ElMAX), Mar Chica lagoon (MCh), and Bizerte lagoon (MIZ), and their expression level. In the case of Genoa, no raw sample material was available, however, because of the possibility of accessing its metagenome library (Martinez-Martinez et al. 2018, ACS Chem Biol 13:225-234, doi: 10.1021/acschembio.7b00996), we also included enzymes retrieved from this site. In all cases, Protein ID, molecular mass, isoelectric point (pI), protein score, number of peptides identified, PSM (Peptide-to-Spectrum Matches), Exponentially Modified Protein Abundance Index (emPAI) and coverage are specifically indicated per each of the proteins; also, the peptides identified per each of the proteins are detailed. The mass spectrometry proteomics data have been deposited to the ProteomeXchange Consortium via the PRIDE partner repository with the dataset identifier PXD039714 and 10.6019/PXD039714. The samples ID are as follows: MFerrer_RM1-7_F118655, AQ; Ferrer_RM2-28_F118660, MCh; MFerrer_K1-7_F118653, GEN; MFerrer_RM1-28_F118659, ElMAX; MFerrer_RM2-7_F118656, BIZ; MFerrer_K2-7_F118647, PRI; MFerrer_K1-28_F118657, ANC; MFerrer_K2-28_F118658, MES.

File Name: **Supplementary Data S3**
Description: List of the polypeptide sequences of the 83 enzymes (PCR-cloned or synthesized) from the Irish Sea-Mediterranean-the Red Sea longitudinal transect and relative accession numbers from NCBI (https://www.ncbi.nlm.nih.gov/)

File Name: **Supplementary Data S4**
Description: List of 150 synthesized enzymes from 56 TARA Ocean locations and relative accession numbers from the Tara Ocean Data repository in The European Nucleotide Archive (https://www.ebi.ac.uk/services/tara-oceans-data).

File Name: **Supplementary Data S5**
Description: List of OTUs positively and negatively correlated with temperatures and presence of OTUs across the three TV levels (LTV, ITV and HTV).
