# Supplementary material for: Enzyme adaptation to habitat thermal legacy shapes the thermal plasticity of marine microbiomes
Source: Nat Commun. 2023 Feb 24;14:1045. doi: 10.1038/s41467-023-36610-0 (PMC9958047; doi:10.1038/s41467-023-36610-0)
Supplement: Supplementary file 9 — Reporting Summary [file 41467_2023_36610_MOESM9_ESM.pdf]

nature research

Corresponding author(s): Prof. Daniele Daffonchio, Prof. Manuel Ferrer

Last updated by author(s): Jan 28, 2023

Reporting Summary

Nature Research wishes to improve the reproducibility of the work that we publish. This form provides structure for consistency and transparency in reporting. For further information on Nature Research policies, see Authors & Referees and the Editorial Policy Checklist .

Statistics

For all statistical analyses, confirm that the following items are present in the figure legend, table legend, main text, or Methods section.

n/a	Confirmed	
☐	☒	The exact sample size (*n*) for each experimental group/condition, given as a discrete number and unit of measurement
☐	☒	A statement on whether measurements were taken from distinct samples or whether the same sample was measured repeatedly
☐	☒	The statistical test(s) used AND whether they are one- or two-sided<br>*Only common tests should be described solely by name; describe more complex techniques in the Methods section.*
☐	☒	A description of all covariates tested
☐	☒	A description of any assumptions or corrections, such as tests of normality and adjustment for multiple comparisons
☐	☒	A full description of the statistical parameters including central tendency (e.g. means) or other basic estimates (e.g. regression coefficient) AND variation (e.g. standard deviation) or associated estimates of uncertainty (e.g. confidence intervals)
☐	☒	For null hypothesis testing, the test statistic (e.g. *F*, *t*, *r*) with confidence intervals, effect sizes, degrees of freedom and *P* value noted<br>*Give P values as exact values whenever suitable.*
☒	☐	For Bayesian analysis, information on the choice of priors and Markov chain Monte Carlo settings
☒	☐	For hierarchical and complex designs, identification of the appropriate level for tests and full reporting of outcomes
☒	☐	Estimates of effect sizes (e.g. Cohen's *d*, Pearson's *r*), indicating how they were calculated

Our web collection on statistics for biologists contains articles on many of the points above.

Software and code

Policy information about availability of computer code

Data collection	We retrieve Mean Annual Temperature (MAT), Mean salinity and pH for the locations studied from Bio-oracle database (https://www.bio-oracle.org/)<br>We retrieved the sequence and environmental parameters for the Tara Ocean Sampling Expedition samples from The European Nucleotide Archive (https://www.ebi.ac.uk/services/tara-oceans-data)
Data analysis	The software used for this manuscript are:<br>Primer 6 + Permanova; R and R Studio, lm() function, ggtree and ggtreeExtra packages; Prism 9.2; HOBO software; Microsoft Excel 2019; AMBER21 software for MD simulations, available at ambermd.org; AlphaFold2-based workflow of ColabFold, available at https://github.com/sokrypton/ColabFold (accessed 22.02.2022); CNA software, available at cpclab.uni-duesseldorf.de; CAVER 3.0.3 PyMOL Plugin, available at https://www.caver.cz/; NCBI BLAST software (DIAMOND v2.0.9 program); ImageJ (version 1.54b); QGIS v3.10; MEGA11; ClustalW; SigmaPlot 13.0; UPARSE v8; QIIME v1.9; Analyst® TF 1.5.1 Software (AB SCIEX) for MS and MS/MS data processing

For manuscripts utilizing custom algorithms or software that are central to the research but not yet described in published literature, software must be made available to editors/reviewers. We strongly encourage code deposition in a community repository (e.g. GitHub). See the Nature Research guidelines for submitting code & software for further information.

Data

Policy information about availability of data

All manuscripts must include a data availability statement. This statement should provide the following information, where applicable:

- Accession codes, unique identifiers, or web links for publicly available datasets
- A list of figures that have associated raw data
- A description of any restrictions on data availability

Accession numbers are indicated in the manuscript. All data needed to evaluate the conclusions reported in the paper are present in the Supplementary Information, Supplementary Data and Source Data files; specifically:

Data showed in Figure 1 are associated with Supplementary Data S1 and Source Data files
Data showed in Figure 2 are associated with Supplementary Data S3 and S4 files
Data showed in Figure 3 are associated with Source Data file
Data showed in Figure 4 are associated with Source Data and Supplementary S5 files
Data showed in Supplementary Figure S2 are associated with Source Data file
Data showed in Supplementary Figure S3 are associated with Supplementary Table S3 and Supplementary Data S2
Data showed in Supplementary Figure S4 and S5 are associated with Supplementary Data S3
Data showed in Supplementary Figure S6 and S7 are associated with Supplementary Data S4
Sequences showed in Supplementary Figure S8 are associated with Supplementary Data S3 and S4
Proteomics data have been deposited in PRIDE (dataset identifier PXD039714 and 10.6019/PXD039714)
Accession codes with their hyperlinks for all shotgun metagenome and sequences encoding enzymes are provided in Supplementary Table S3, Supplementary Data S3 and S4
Sequencing data of environmental and microcosms microbial communities have been deposited in NCBI database under the SRA accession number PRJNA508596 (https://www.ncbi.nlm.nih.gov/bioproject/PRJNA508596)

Field-specific reporting

Please select the one below that is the best fit for your research. If you are not sure, read the appropriate sections before making your selection.

☐ Life sciences ☐ Behavioural & social sciences ☒ Ecological, evolutionary & environmental sciences

For a reference copy of the document with all sections, see nature.com/documents/nr-reporting-summary-flat.pdf

Ecological, evolutionary & environmental sciences study design

All studies must disclose on these points even when the disclosure is negative.

Study description

The temperature-dependent response of active proteins extracted from marine sediment microbial communities was conducted by sampling along a north-south latitudinal gradient spanning from the Irish Sea to the Southern Red Sea (14 locations). The optimum of temperature was evaluated by quantifying the thermal activity of seven functionally-independent enzyme classes (esterase, extradiol dioxygenase, phosphatase, beta-galactosidase, nuclease, transaminase, and aldo-keto reductase). To validate the thermal pattern observed, the activity and stability profiles of 228 esterases (78 from the transect described above and 150 from Tara Ocean Expedition dataset) and 5 extradiol dioxygenases (from the transect above) across 70 locations worldwide (latitude range 62.2°S–16° N, MAT range, from –1.4ºC to 29.5ºC) was performed.

In addition, we explore the responses of esterases to different thermal regimes when the MAT does not differ significantly in the sediment/locations. We selected three Red Sea coastal locations. Each station, exposed to a different range of temperature variation (TV), was sampled in triplicate for a total of nine sampling sites and 27 sediment samples. Two seasons were considered, August and December, for a total of 54 samples. Our purpose was to assess how the thermal response of microbial community is linked to the selection of enzymes adapted to work under specific thermal variation.

Our findings show how marine microbial communities exposed to wider thermal variation are able to better cope with temperature variations and their response mechanisms are globally consistent, leading us to conclude that the adaptation capacity of microbial communities to cope in different thermal variation should be taken in consideration when modelling the microbial response in future climate change scenario.

Research sample

The rationale for the sample choice was to analyze marine sediments and seawaters from locations with different mean annual temperature (MAT) and thermal variations (TV). A north-south transect spanning from the Irish Sea to the Southern Red Sea was chosen for marine sediment sampling, along with a series of metagenomes from the Tara Ocean seawater sequence datasets from different locations to cover a large set of MATs. Please see also the "study description" section above.

1. Across the latitudinal transect from the Irish Sea to the Southern Red Sea sediment samples were obtained from 14 locations (n=3 per location), corresponding to a gradient of different MAT. Location details and MAT are summarized in Supplementary Table S1. The native proteins extracted from sediment as bulk were used to quantifying the thermal behaviour (optimal temperature for enzyme activity) of seven functionally-independent enzyme classes (esterase, extradiol dioxygenase, phosphatase, beta-galactosidase, nuclease, transaminase, and aldo-keto reductase).

2. From 10 locations of the transect described above, 78 esterases and 5 extradiol dioxygenase were retrieved and cloned/synthesized to evaluate their thermal characteristics, that are temperature for optimal activity (Topt), stability by meaning of denaturing temperature (Td) measured by circular dichroism and flexibility by meaning of the computed phase transition temperature (Tp), a measure for global structural rigidity.

3. To enlarge the explored MAT, 150 esterase sequences were retrieved from the Tara Ocean dataset available (from The European Nucleotide Archive; https://www.ebi.ac.uk/ena/browser/view/ERS491095). The selected enzymes were also synthesized to evaluate the same thermal characteristics detailed before at point 2.

4. To evaluate the "tuning" effect of thermal variation (TV) in site with similar MAT sediment samples from nine locations were collected in triplicate, both in August and December for a total of 54. The growth of microbial communities (i.e., enriched heterotrophic bacteria) extracted from the sediment, for each station, was tested at 5 different temperatures in 8 replicates. Therefore, the growth of enriched heterotrophic bacteria was tested in a total of 72 replicates at each of the five temperature. The total active proteins were extracted from the corresponding Red Sea sediments and tested for their activity/stability at different temperature, along with the DNA in order to sequence 16S rRNA gene to evaluate bacterial diversity.

Sampling strategy

Sediments along the coastlines of the Irish Sea, the Mediterranean Sea, and the Red Sea (from 16°N to 53°N) were sampled applying uniform sampling and storage procedures; 5 Kg of sediments were collected in triplicates by scuba.

Additional sediment samples from Red Sea to evaluate effect of thermal variation (TV) were collected using a Van der Venn grab

system equipped on the research vessel "Explorer" provided by the Coastal and Marine Resource core lab (CMR) at King Abdullah University of Science and Technology (KAUST, Saudi Arabia). Only the first 5-10 centimeters of the sediments were collected in sterile tubes avoiding the perturbed sediments close to the sides of the grab system.

Data collection

Samples from the different locations of the latitudinal transect from the Irish Sea to the Southern Red Sea were collected by collaborative partners within the EU project ULIXES (reference 266473) who delivered the samples to Manuel Ferrer (CSIC). Samples from the Irish Sea and the preparation of enrichments and their sequencing were delivered by Bangor (Rafael Bargiela, Tatyana N. Chernikova, Peter N. Golyshin). Samples from the Central and the Southern Red Sea were recovered by the Authors RM, MFu, AB and DD. The gene/protein sequences from the Tara Ocean datasets available were recovered by Manuel Ferrer and Sergio Sánchez-Carrillo (ICP, CSIC). The enzymatic analysis (Topt and Td) were conducted at the ICP-CSIC, and at the Spectroscopy Laboratory, Centro de Investigaciones Biológicas (CIB-CSIC). Analysis of flexibility was conducted by Christina Gohlke neé Nutschel, Christopher Pfleger, Jonas Dittrich and Holger Gohlke (IBG-4, Heinrich-Heine-Universität Düsseldorf, NIC-JSC, Germany). Environmental data of studied locations were obtained from in situ-measurement and Bio-oracle database (https://www.bio-oracle.org/). The Red Sea Time Series were obtained by deploying 3 HOBO temperature loggers at each of the three depths sampled (HTV, ITV and LTV).

Timing and spatial scale

1. The rationale of sampling across a latitudinal scale from the Irish Sea to the Southern Red Sea was for getting microbiomes and enzymes from sites with different mean annual temperatures (MAT) and test the thermal response of the enzymes. A total of 14 locations between the Irish Sea, Mediterranean Sea and Red Sea were collected in triplicates avoiding sediment perturbation between the triplicates.
2. The choice of the timing scale for sampling (2 seasons) in the Central Red Sea was to assess the thermal response of the sediment microbiomes and enzymes in the coldest as well as the warmest seasons in the Central Red Sea, while the choice of the spatial scale (3 sites) was to obtain sediment microbiomes from sites with the same mean annual temperature but with different thermal variability (high in the shallow sediments and low in the deeper sediments). Specifically, coastal sediment samples were collected in the Red Sea in August and December 2015. Seawater temperature above the sediments was recorded from March 2015 and September 2016. The sampling stations were divided in three replicates for three different depths that were experiencing different thermal regimes. The stations were spatially divided to avoid perturbation due to the sampling operations.

Data exclusions

The sediment sample from the Gulf of Genoa was not used for activity tests and metaproteome analysis because no raw sample material was available; however, because of the possibility to access to its shotgun metagenome and a metagenome clone library, we used both bio-resourced to recover esterases to incorporate an additional latitude in our transect. This has been mentioned in the manuscript.

Reproducibility

In total we included in our experiments 14 locations in a large latitudinal range and 9 locations in Central Red Sea, observing the same pattern for enzymatic thermal response. All the stations are easily accessible to perform again the sampling and the analysis conducted in this study. Moreover, we collected Red Sea coastal sediments in other seasons over August and December and in different years, observing the same trends in the microbial community response to the seasonal temperature and in the manipulative experiments performed in the laboratory. The enzymatic activity was performed including enzymes isolates from 10 different locations and the dataset was further validated including 150 enzymes from 56 Tara Ocean Expedition sites that have similar sequence to the one isolated in our studies. The consistency of the results obtained on a worldwide scale indicaes that our experiments are reproducible also on samples collected in different sites from the ones chosen in our dataset.

Randomization

We chose locations along a broad climate gradient, but not in a manner that controlled potential influential variables other than temperature. Samples from each station were collected multiple time keeping the same area of sampling (i.e., same sediments depth, same coordinates), but the sediments were randomly collected avoiding area previously perturbed by the sampling. This strategy increases the area of the sampling maintaining the environmental characteristics of the sample, but increasing the randomization factors of any sample improving the reliability of the generated dataset.

Blinding

Perform blind experiments was not the purpose of our analysis. Moreover, the same operators conducted different analysis on the same samples making impossible the blind approach since they needed to know the origin of the samples to correctly set the replicates and the incubation protocols. However, the enzymatic analysis were conducted in Spain with other collaborators that received the samples with a code name where was not specified the sediment location. Therefore, these experiments were partially performed blindly.

Did the study involve field work? ☒ Yes ☐ No

Field work, collection and transport

Field conditions

Sediment sampling was conducted in different locations and in different seasons to capture temporal variation in microbial community attributes. Since we were interested in the community associated to the surface part of the sediments, we collected samples to previous unperturbed spots. The collected samples were rapidly moved in the laboratory and processed straight forward after the collection.

Location

Red Sea sediments exposed to different thermal variations (TV) were collected between the following coordinates range: 22° 16'34.44"N - 22°19'40.23"N, 39°1'34.65"E - 39°5'8.30"E. The other locations included in this study are the following: Menai Strait (Ireland): 53°13'32.00"N, 4°9'35.00"W; Ancona harbor (Italy): 43°37'0.00"N, 13°50'15.00"E; Milazo harbor (Italy): 38°12'30.10"N, 15°15'34.89"E; Messina harbor (Italy): 38°11'42.27"N, 15°34'25.01"E; Priolo Gargallo harbor (Italy): 37°16'8.90"N, 9°53'20.10"E; Bizerte lagoon (Tunisia): 37°10'27.46"N, 15°12'7.50"E; Mar Chica lagoon (Marocco): 31°9'31.20"N, 29°50'28.20"E; El-Max (Egypt): 35°11'57.10"N, 2°55'37.60"W; Gulf of Aquaba (Jordan): 30°22'0.42"N, 25°24'57.00"E; Alkarar lagoon (Saudi Arabia): 22° 57'29.94"N, 38°52'20.88"E; KAEC, Thuwal (Saudi Arabia): 22°24'28.02"N, 39°7'58.44"E; Farasan island (Saudi Arabia): 16° 52'40.02"N, 42°10'35.46"E; Jizan(Saudi Arabia): 16°45'5.22"N, 42°2'17.94"E and Jizan (Saudi Arabia): 16°45'5.22"N, 42° 2'17.94"E. Location of the 150 esterases retrieved from TARA Ocean dataset are reported in Supplementary Data S4.

Access and import/export

Permission and/or permits were managed in compliance with the KAUST policy and issued by the Saudi coast guard with the help of Coastal and Marine Resource core lab (CMOR, KAUST, Saudi Arabia). Transfer of sediment material from KAUST to Spain was

	regulated by a material transfer agreement between KAUST (Saudi Arabia) and CSIC (Spain), issued by KAUST.
Disturbance	The Van der Venn grab system used to collect the sediments affect 1 dm3 of the sediment surface. We carefully avoid sediments close to reef area to minimize disturbance to sensitive ecosystems.

Reporting for specific materials, systems and methods

We require information from authors about some types of materials, experimental systems and methods used in many studies. Here, indicate whether each material, system or method listed is relevant to your study. If you are not sure if a list item applies to your research, read the appropriate section before selecting a response.

Materials & experimental systems

n/a	Involved in the study
☒	☐ Antibodies
☒	☐ Eukaryotic cell lines
☒	☐ Palaeontology
☒	☐ Animals and other organisms
☒	☐ Human research participants
☒	☐ Clinical data

Methods

n/a	Involved in the study
☒	☐ ChIP-seq
☒	☐ Flow cytometry
☒	☐ MRI-based neuroimaging
